# Supplementary material for: Structural insights into proprotein convertase activation facilitate the engineering of highly specific furin inhibitors
Source: Nat Commun. 2025 Sep 2;16:8206. doi: 10.1038/s41467-025-63479-y (PMC12405487; doi:10.1038/s41467-025-63479-y)
Supplement: Supplementary file 1 — Supplementary Information [file 41467_2025_63479_MOESM1_ESM.pdf]

SUPPLEMENTARY INFORMATION

Structural insights into proprotein convertase activation facilitate the engineering of highly specific furin inhibitors.

Rupert Klaushofer[1,2‡], Konstantin Bloch[3‡], Luisa Susanna Eder[1,2], Simone Marzaro[1], Mario Schubert[1], Eva Böttcher-Friebertshäuser[3], Hans Brandstetter[1,2] and Sven O. Dahms[1,2,*]

[1] Department of Biosciences and Medical Biology, University of Salzburg, Hellbrunner Straße 34, A-5020 Salzburg, Austria

[2] Center for Tumor Biology and Immunology (CTBI), University of Salzburg, Hellbrunner Straße 34, A-5020 Salzburg, Austria

[3]Institute of Virology, Philipps University, Hans-Meerwein-Straße 2, D-35043 Marburg, Germany

[‡] Authors contributed equally

* To whom correspondence should be addressed:

Sven O. Dahms: sven.dahms@plus.ac.at, Tel: +43-662-80447277

Table of contents

	Page
Supplementary Methods	3-5
Supplementary Figures	
Figure S1	6
Figure S2	7
Figure S3	8
Figure S4	9
Figure S5	10
Figure S6	11
Figure S7	12
Figure S8	13
Figure S9	14
Figure S10	15
Figure S11	16
Figure S12	17
Figure S13	18
Figure S14	19
Figure S15	20
Figure S16	21
Figure S17	22
Figure S18	23
Supplementary Tables	
Table S1	24
Table S2	25
Table S3	26
Table S4	27
Supplementary References	28

Supplementary Methods

Expression and purification of proprotein convertases.

The expression plasmids for furin (Asp23-Ala574, UNP P09958 [1-3]), furin^{T562R} (Asp23-Ala574 with Thr562Arg-mutation, UNP P09958 [4]), PC5/6 (Arg33-Gly634, UNP Q92824, [1]) and PC7 (Pro44-Thr667, UNP Q16549, [1]) were synthesized and cloned by GeneArt (Thermo Fisher Scientific). In all cases, the DNA was codon-optimized for *Homo sapiens*. The constructs were modified at the 5'-end (TGATCAGCCACCATGGGGATTCTTCCCAGCCCTGGGATGCCTGCGCTGCTCTCCCTCG TGAGCCTTCTCTCCGTGCTGCTGATGGGTTGCGTAGCTGAAACCGGT) including the secretion signal sequence and a Bcl-I restriction site. At the 3'-end the sequences of furin (wild-type) and of PC5/6 as well as PC7 were extended by AGCGGTAGCCTGGTGCCGCGCGGCAGCCACCACCATCACCACCACTGATGACTCGAG and GGATCCCACCACCATCACCACCACTGATAATCACTCGAG, respectively. The DNA sequences of furin (wild-type), PC7 and PC5/6 were inserted into the pCDNA3.1(+) expression vector (Invitrogen, Thermo Fisher Scientific) using BclI and XhoI for the insert-DNA fragment as well as BamHI and XhoI for the vector-DNA fragment. The 5'- and 3'-ends of the furin^{T562R} coding sequence were modified with ACCGGT and AGCGGTAGCCTGGTGCCGCGCGGCAGCCACCACCATCACCACCACTGA TGACTCGAG. The modified DNA sequence of furin^{T562R} was inserted in the expression vector pHLsec [5] using the restriction enzymes AgeI and XhoI. The pCDNA3.1(+)-derived plasmids were linearized with ScaI (FastDigest, Thermo Fisher Scientific) and used for stable transfection of HEK293S (ATCC CRL3022) cells [1].

Cells were grown in DMEM (4.5 g l^{-1} glucose, 2 mM L-glutamine, 110 mg L^{-1} sodium pyruvate, 3.7 g L^{-1} $NaHCO_3$, nonessential amino acids; PAN-Biotech GmbH) supplemented with 10% (v/v) FBS (Qualified Standard Origin Brazil, Gibco, Thermo Fisher Scientific). Polyclonal selection of stable cell lines was conducted in the presence of 500 µg mL^{-1} G418 (Geneticin, Carl Roth GmbH). Large-scale protein expression was performed in multilayer flasks (HyperFlask M, Corning) without G418. For protein expression the medium of confluently grown cells was exchanged with DMEM supplemented with 2 mM sodium butyrate as well as with FBS (2% (v/v), furin) or without FBS (PC5/6 and PC7). The medium exchange was repeated five (furin) or two (PC5/6 and PC7) times every 2-3 days. After harvest the conditioned medium was centrifuged (20 min, 5500 g, 4 °C) and stored at −80 °C until further use. Furin^{T562R} was expressed by transient expression of HEK293S [2,3] in 175 cm^2 cell culture flasks (CELLSTAR TC, Red filter screw cap, Greiner Bio-One) with 2% (v/v) FBS, and 5 mM $CaCl_2$ according to the procedure described by Aricescu and coworkers [5]. The ratio (w/w) of transfection reagent to plasmid-DNA was 2:1 and 0.5 µg DNA were used per cm^2 of cultured cells. The medium was exchanged 24 h after transfection by DMEM without FBS but supplemented with 2 mM CaCl2. After 72 h the conditioned medium was harvested, centrifuged and stored as described above.

Before purification, the medium was thawed in a water bath at 37°C and concentrated by ultrafiltration (Omega 10K ultrafiltration membrane, Pall) in a tangential flow concentrator (Centramate, Pall). During concentration the medium was cooled on ice. Purification steps were performed at ~22°C if not stated otherwise. Concentrated medium was diluted 1:5 in buffer A (100 mM Tris/HCl, pH 8.0, 500 mM NaCl), concentrated again and filtered using a 0.22 µm membrane (GP Millipore Express PLUS, Millipore). Prior to immobilized metal affinity purification (IMAC) buffer B (buffer A supplemented with 500 mM imidazol) was added to the medium to reach a concentration of 10 mM imidazole. NiNTA-superflow resin (Qiagen) was equilibrated in buffer A + 10 mM imidazole, added to the medium and incubated for 30 min.

The suspension was loaded on a gravity flow column jacket, washed three times with 10 bed volumes (cv) of buffer A + 10 mM imidazole and eluted four times with 1 cv buffer B.

Furin (wild-type and Thr562Arg) was further purified using immobilized inhibitor affinity purification [6]. For this purpose, the IMAC elution fractions with the highest protein content were pooled, diluted 1:5 with 10 mM Hepes/NaOH, pH 7.4, 2 mM $CaCl_2$ and loaded on the inhibitor affinity column. The protein was eluted with 10 mM Hepes/NaOH, pH 7.4, 500 mM NaCl and 2 mM $CaCl_2$, diluted 1:5 with 10 mM Hepes/NaOH, pH 7.4, 2 mM $CaCl_2$ and concentrated by ultrafiltration (Amicon Ultra 15 Centrifugal Filter Ultracel 30K, Merck Millipore).

PC7 was diluted in 50 mM Tris/HCl, at pH 7.5, 10 mM $CaCl_2$, and 0.15 M NaCl to 50 µg mL^{-1} and activated with thermolysin (2.0 µg mL^{-1}) for 16 h at 37 °C. The reaction mixture was concentrated by ultrafiltration (Amicon Ultra 15 Centrifugal Filter Ultracel 30K, Merck Millipore) and subjected to gel permeation chromatography (GPC, both steps at 4 °C).

IMAC fraction of PC5/6 were concentrated by ultrafiltration (Amicon Ultra 15 Centrifugal Filter Ultracel 30K, Merck Millipore) and subjected to GPC. For PC5/6 and PC7 GPC was performed on a Superdex 200 10/300 GL column (GE Healthcare) coupled to a coupled to a chromatography system (Aekta Purifier with Unicorn 5.31 software, GE Healthcare) using 10 mM Hepes/NaOH, pH 7.4, 100 mM NaCl and 2 mM $CaCl_2$. Fractions with high protein content were pooled and concentrated by ultrafiltration.

Cloning of PC1/3-prodomain constructs.

The expression vectors covering the amino acids 28-110 of human PC1/3 (PCSK1, UNIPROT-ID P29120) were synthesized and cloned by GeneArt (Thermo Fisher Scientific, wild-type PC1/3-prodomain, **M1**-**M5**, His_6-**M5**, **M9** and **M10)**. In all cases the coding sequences (CDS) were codon-optimized for *E. Coli*. The CDS of the wild-type PC1/3 prodomain, **M2**, **M3**, **M9** and **M10** were modified at the 5’-end with the sequence CCATGGGCCATCATCATCATCATCACTCTGGTACCGAAAATCTGTATTTTCAGGGT adding a NcoI restriction site, the His-tag sequence and the TEV cleavage site. The 3’-end of the CDS was modified with the sequence TGATAGCTCGAG that contains a XhoI restriction site. The insert sequences for **M1**, **M4**-**M8** and wild-type PC1/3-prodomain. For the wild-type protein a second construct type were generated (that essentially lead to the same protein after TEV-cleavage and is expressed and purified in the same way) containing from the 5’-end to the 3’-end the sequence CCATGGGCCATCATCATCATCATCACTCTGGCCAT (including a NcoI restriction site and the His-tag sequence), the DNA sequence covering the amino acids 25-260 of the Uniprot entry A0A1S4NYF2 (mNeonGreen), the sequence TCTGGTACCGAAAATCTGTATTTTCAGGGTAA coding for the TEV-cleavage site, the sequence of the PC1/3 prodomain and the sequence TGATAGCTCGAG that contains a XhoI restriction site. The CDS of His_6-**M5** was modfied at the 5’- and 3’-end with the sequences CCATGGGCCATCATCA TCATCATCACTCTGGCCATATG (NcoI restriction site and His-tag) and TGATAGCTCGAG (XhoI restriction site), respectively. The DNA sequences of all PC1/3-prodomain variants were inserted in the pET28a(+) expression vector (Novagen) between the NcoI and XhoI restriction sites. PC1/3 prodomain constructs **M6**, **M7** and **M8** (His72 mutants of the wild-type PC1/3-prodomain) were generated by site-directed mutagenesis using the primer pairs GTTCACTTGAAAATCACTACTTATTCAAAAATAAAAACCACCCCAGAAGGTC (forward) and GACCTTCTGGGGTGGTTTTTATTTTTGAATAAGTAGTGATTTTCAAGTGAAC (reverse), GTTCACTTGAAAATCACTACTTATTCAAAGATAAAAACCACCCCAGAAGGTC (forward) and GACCTTCTGGGGTGGTTTTTATCTTTGAATAAGTAGTGATTTTCAAGTGAAC (reverse) as well as GTTCACTTGAAAATCACTACTTATTCAAACTGAAAAACCACCCCAGAAGGTC (forward)

and GACCTTCTGGGGTGGTTTTTCAGTTTGAATAAGTAGTGATTTTCAAGTGAAC (reverse), respectively.

Cloning of nanobody-PC1/3-prodomain fusion constructs.

The expression vectors covering the amino acids 3-119 of the nanobody Nb14 (PDB-ID 5JMR, [4]) and the amino acids 29-110 of the PC1/3-prodomain mutants **M5** and **M9** were synthesized and cloned by GeneArt (Thermo Fisher Scientific). In all cases the coding sequences (CDS) were codon-optimized for *E. Coli*. The insert sequences contained from the 5'-end to the 3'-end the sequence CATATGAAATACCTGCTGCCGACCGCTGCTGCTGGTCTGCTGCTCCTCGCTGCCCAGCCGGCCATGGCCCAGGGTAGCCATCACCATCATCATCATAGCGGTGAAAATCTGTATTTTCAGGGTAGT (NdeI restriction site, pelB leader sequence, His-tag and TEV-cleavage site), the CDS of Nb14, GGTCAGGTTACTAGTGCCGGTGCTAGCGGTCAG (a linker sequence) and the sequence TGATAACTCGAG (XhoI restriction site). The DNA sequences of all PC1/3-prodomain variants were inserted in the pET28a(+) expression vector (Novagen) between the NdeI and XhoI restriction sites.

Cloning of the nanobody Nb14

The expression vector of the nanobody Nb14 covering the amino acids 29-106 of the PC1/3-prodomain mutant **M9** and the amino acids 3-119 of the nanobody Nb14 (PDB-ID 5JMR, [4]) was synthesized and cloned by GeneArt (Thermo Fisher Scientific). The coding sequences (CDS) were codon-optimized for *E. Coli*. The insert sequences contained from the 5'-end to the 3'-end the sequence CATATGAAATACCTGCTGCCGACCGCTGCTGCTGGTCTGCTGCTCCTCGCTGCCCAGCCGGCCATGGCCCAGGGTAGCCATCACCATCATCATCATAGCGGTGAAAATGCATTTTATAATGGTGGTCAG (NdeI restriction site, pelB leader sequence, His-tag, linker), the CDS of the PC1/3-prodomain mutant **M9**, the sequence AATCTGTATTTTCAGGGTAGT (linker and TEV-cleavage site), the CDS of Nb14 and the sequence TGATAACTCGAG.

Supplementary Figures

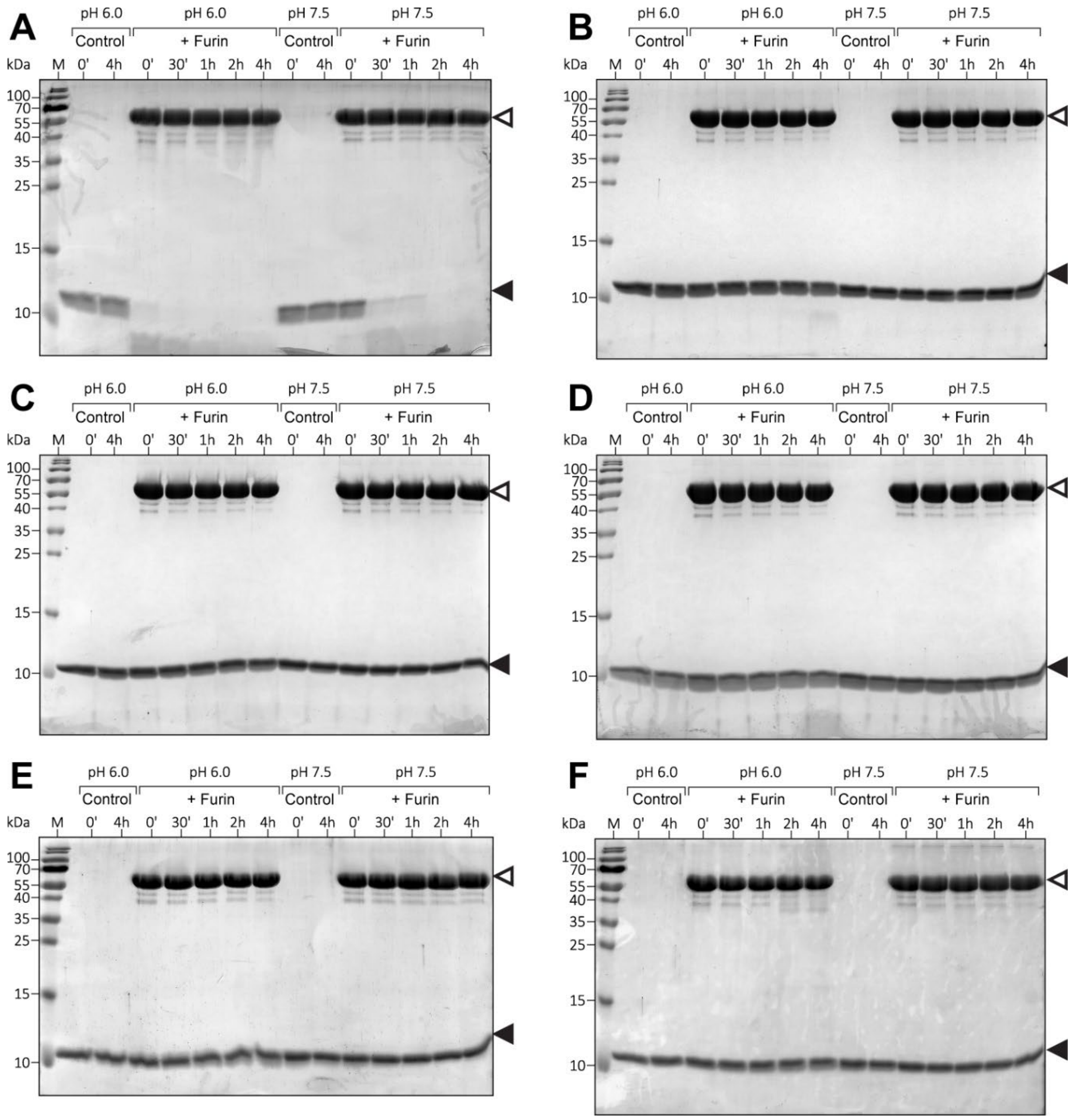

Figure S1 Limited proteolysis assays of PC1/3-prodomain variants. SDS-PAGE analysis limited proteolysis assays of A) the wild-type PC1/3-prodomain as well as mutants B) **M1**, C) **M2**, D) **M3**, E) **M4** and F) **M5**. Assays were either performed with (+Furin) or without furin (Control). The bands of furin and of the PC1/3-prodomain are highlighted by open and closed arrowheads, respectively.

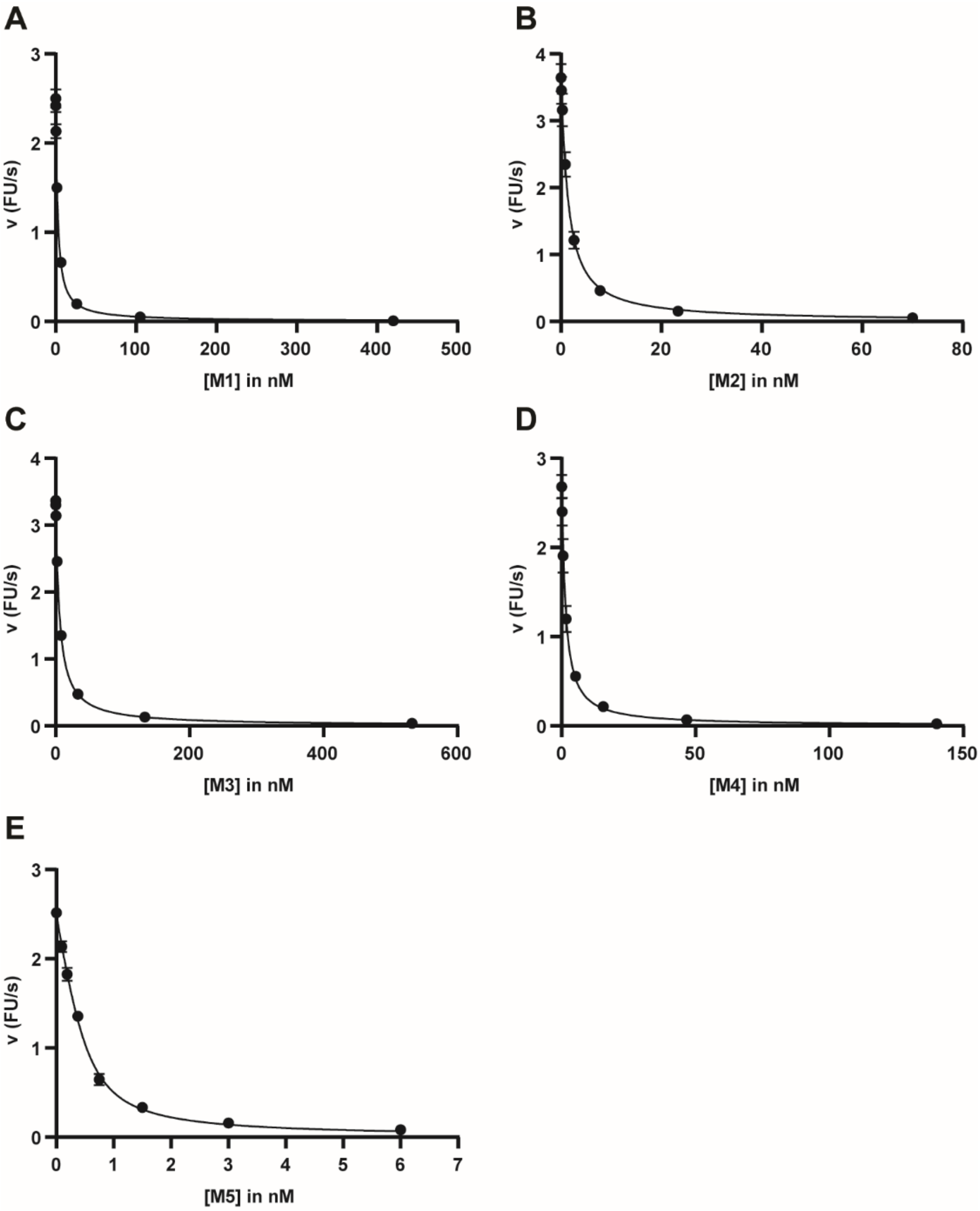

Figure S2 Inhibition of furin by the PC1/3-prodomain mutants A) **M1**, B) **M2**, C) **M3**, D) **M4** and E) **M5** under tight binding conditions. The fit of the points with the Morrison equation is always shown as line. Error bars represent the standard deviations of three replicates (n=3).

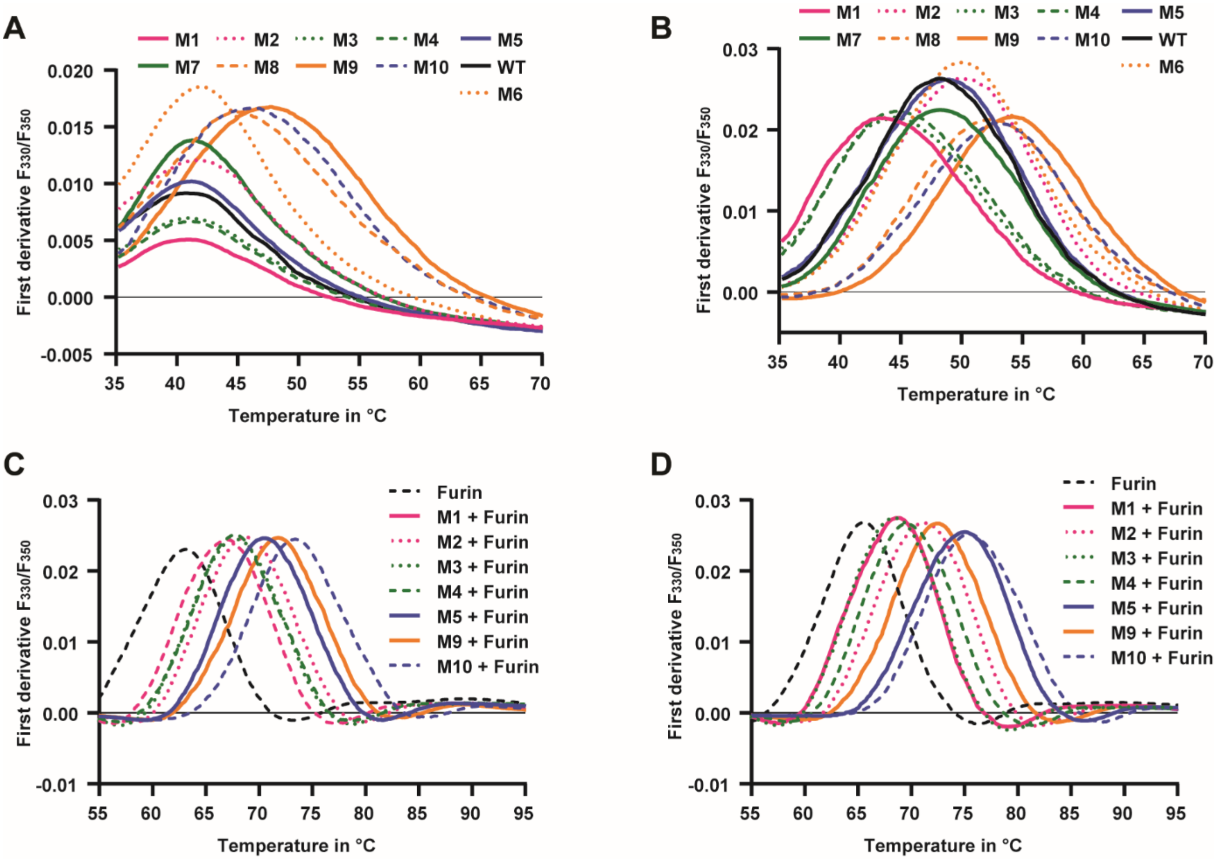

Figure S3 First order derivatives of the melting curves of PC1/3-prodomain variants at A) pH 6.0 and B) 7.4 as well as of furin:PC1/3-prodomain complexes at C) pH 6.0 and D) pH 7.4. (M = mutant, WT = wild-type)

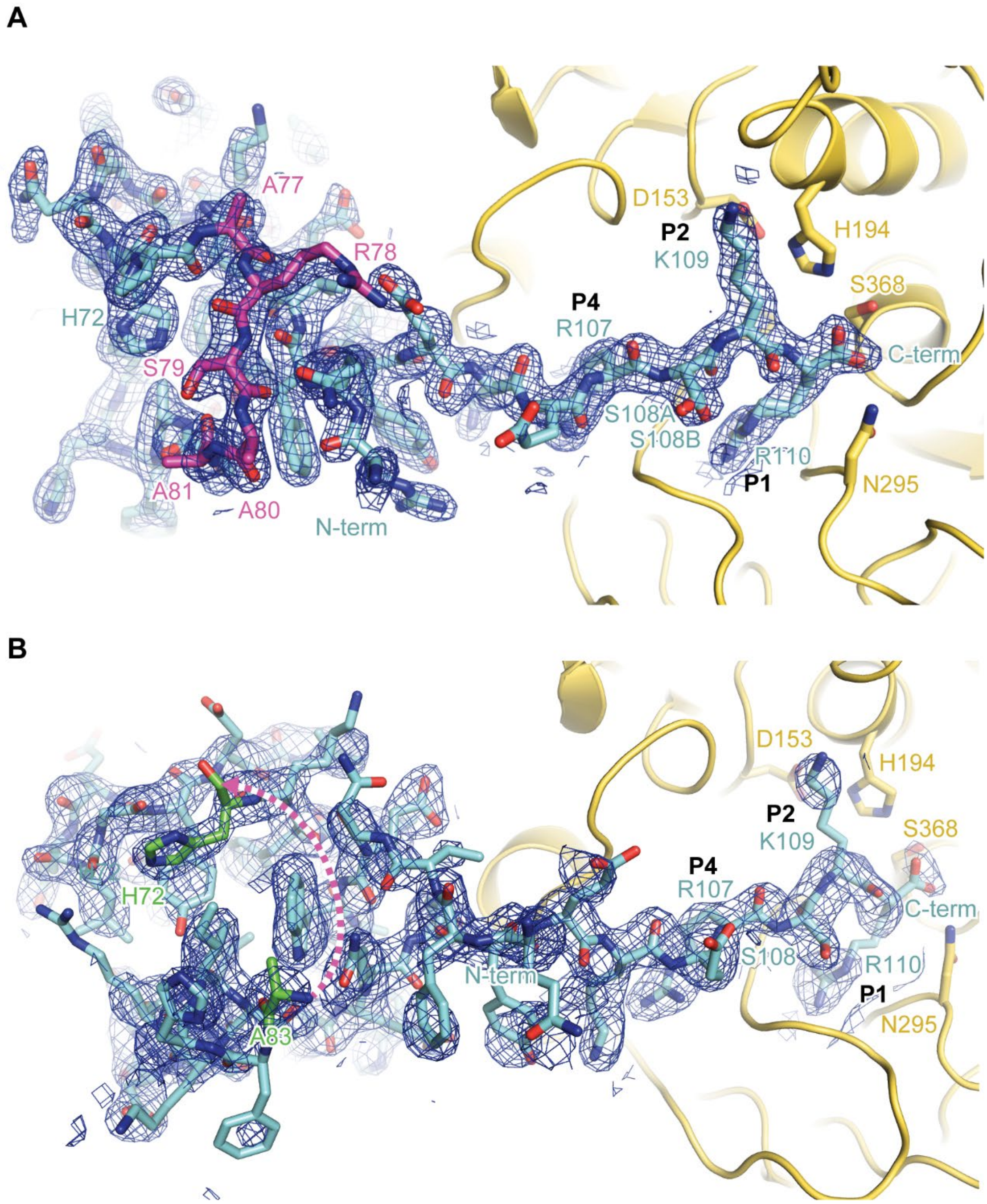

Figure S4 Structure of the PC1/3-prodomain mutants A) **M2** (pH 7.0) and B) **M5** (pH 5.9) bound to furin. Furin (standard orientation) is colored in yellow (cartoon) and the active site residues are shown as stick model. The prodomain is shown as stick model (cyan). The composite $2\boldsymbol{F}_o$-$\boldsymbol{F}_c$ omit electron density map is contoured at 1 σ (blue mesh). The canonical interacting C-terminal residues 107-110 (P1-P4) are marked. A) The secondary cleavage site loop is marked in magenta. Note that this loop of **M2** carries the sequence 77-ARSAA instead of the wild-type sequence 77-RRSRR. B) No electron density of the secondary cleavage site loop of **M5** was observed at pH 5.9. The flanking residues His72 and Ala83 of this loop, which still revealed a defined electron density are marked in green. Thus, the secondary cleavage site loop residues could not be modeled, and the dashed, magenta-colored arrow indicates the chain break between His72 and Ala83.

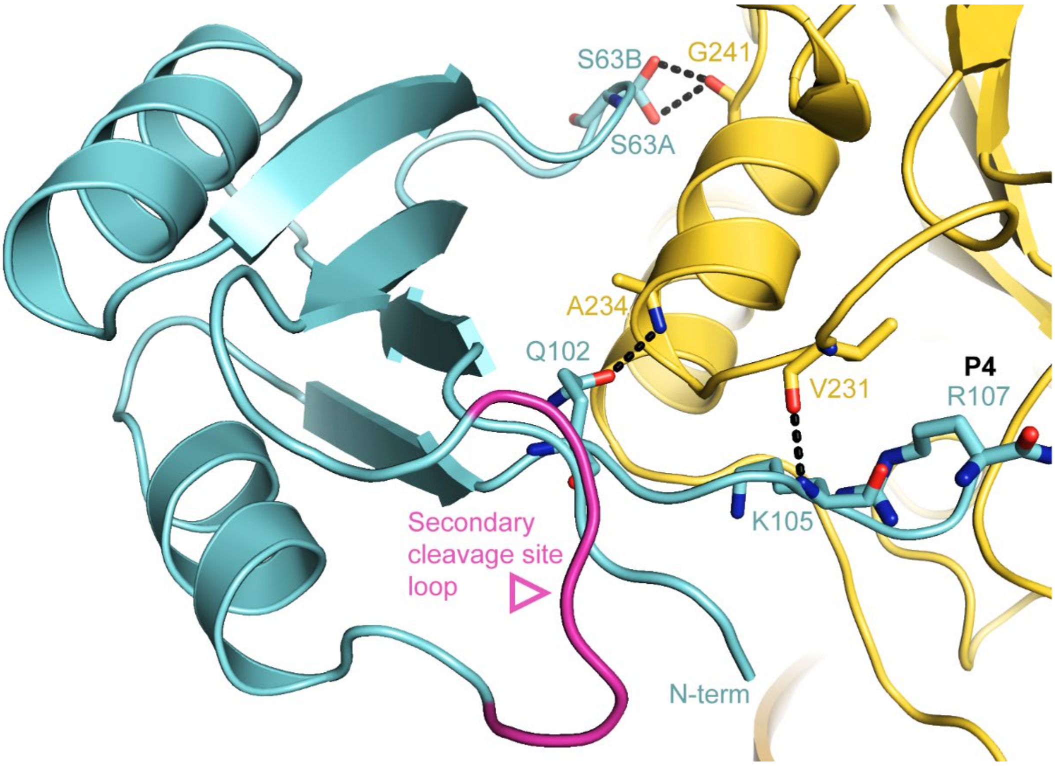

Figure S5 Interactions of the PC1/3-prodomain with furin. The structure of **M2**:furin is shown as cartoon. The prodomain and Furin (in standard orientation) are colored in cyan and yellow, respectively. The secondary cleavage site loop is marked in magenta. Hydrogen bonds are marked by dashed lines. Important residues are given as stick model. S62 was modeled in alternative conformations (S62A and S62B).

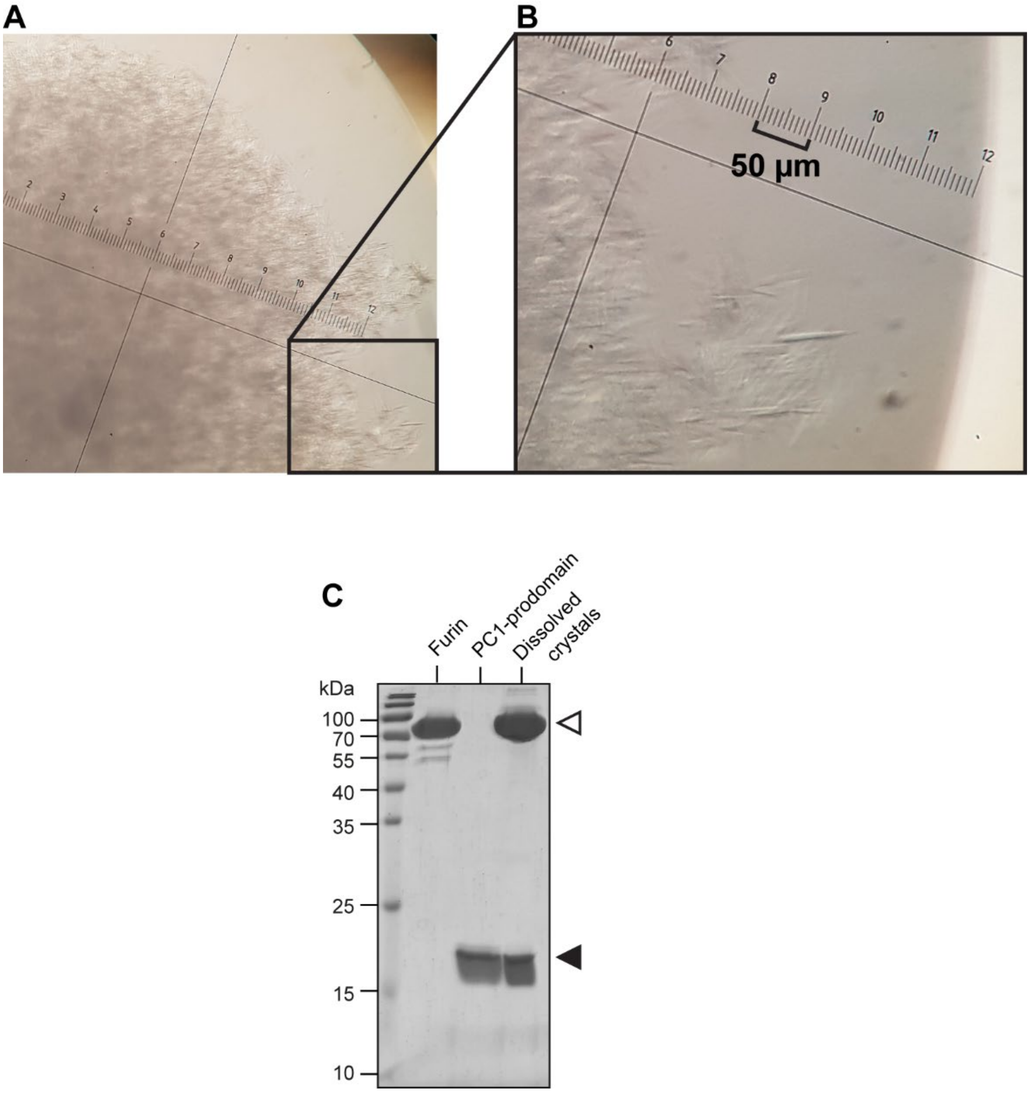

Figure S6 Microcrystals of the **M5**:furin complex. A) Overview picture of a typical crystallization drop. B) Microcrystals at higher magnification with 50 µm scale indicated. C) SDS-PAGE analysis of dissolved microcrystals in comparison to the furin and M5-preparations used for preparation of the crystallization drop. The bands of furin and of the PC1/3-prodomain are highlighted by open and closed arrowheads, respectively.

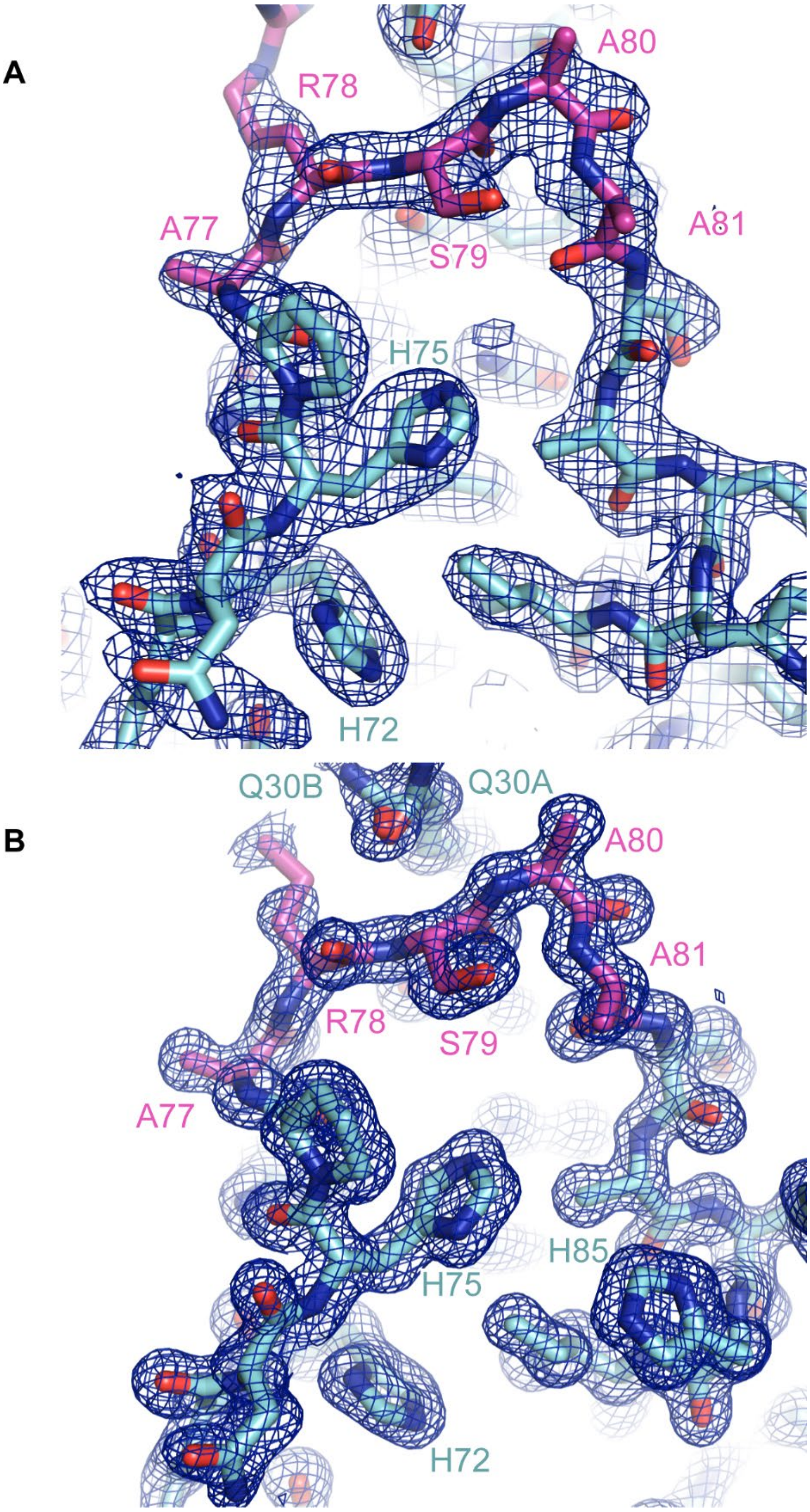

Figure S7 Structure of the secondary cleavage site and flanking regions of A) isolated **M2** (M = mutant) and B) **M2** bound to furin. The prodomain is shown as stick model (cyan) and the composite $2\boldsymbol{F}_o$-$\boldsymbol{F}_c$ omit electron density map is contoured at 1 σ (blue mesh). Note that the secondary cleavage site loop of **M2** carries the sequence 77-ARSAA instead of the wild-type sequence 77-RRSRR.

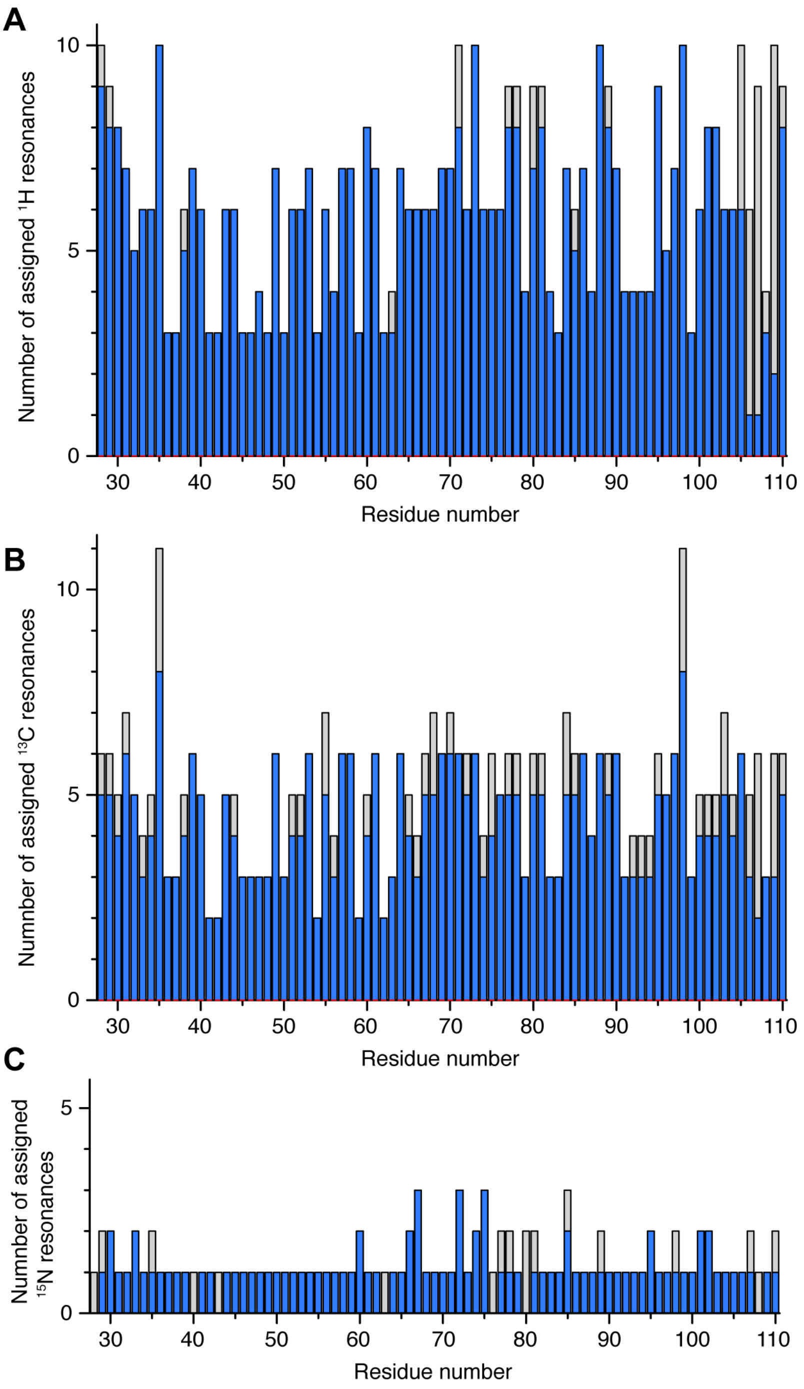

Figure S8 Completeness of the chemical shift assignments for ^{1}H, ^{13}C and ^{15}N nuclei. The chemical shift assignments are rather complete except some residues in the C-terminus (^{1}H, ^{13}C and ^{15}N), some quaternary carbons, which are quite often not assigned and irrelevant for NOE assignments and some ^{15}N chemical shifts, either due to the lack of amide protons (Pro) or non-observable signals in ^{1}H-^{15}N correlations (Ser63, Arg80, His85 and Ser108) and hard to assign side chain signals e.g. Nε of Arg. A) Number of assigned ^{1}H chemical shifts per residue (blue) and maximal observable chemical shifts per residue (grey). B) Number of assigned ^{13}C chemical shifts per residue (blue) and maximal observable chemical shifts per residue (grey). C) Number of assigned ^{15}N chemical shifts per residue (blue) and maximal observable chemical shifts per residue (grey). Chemical shifts of Nζ of arginines were not included, because their correlations are typically not observable at neutral conditions.

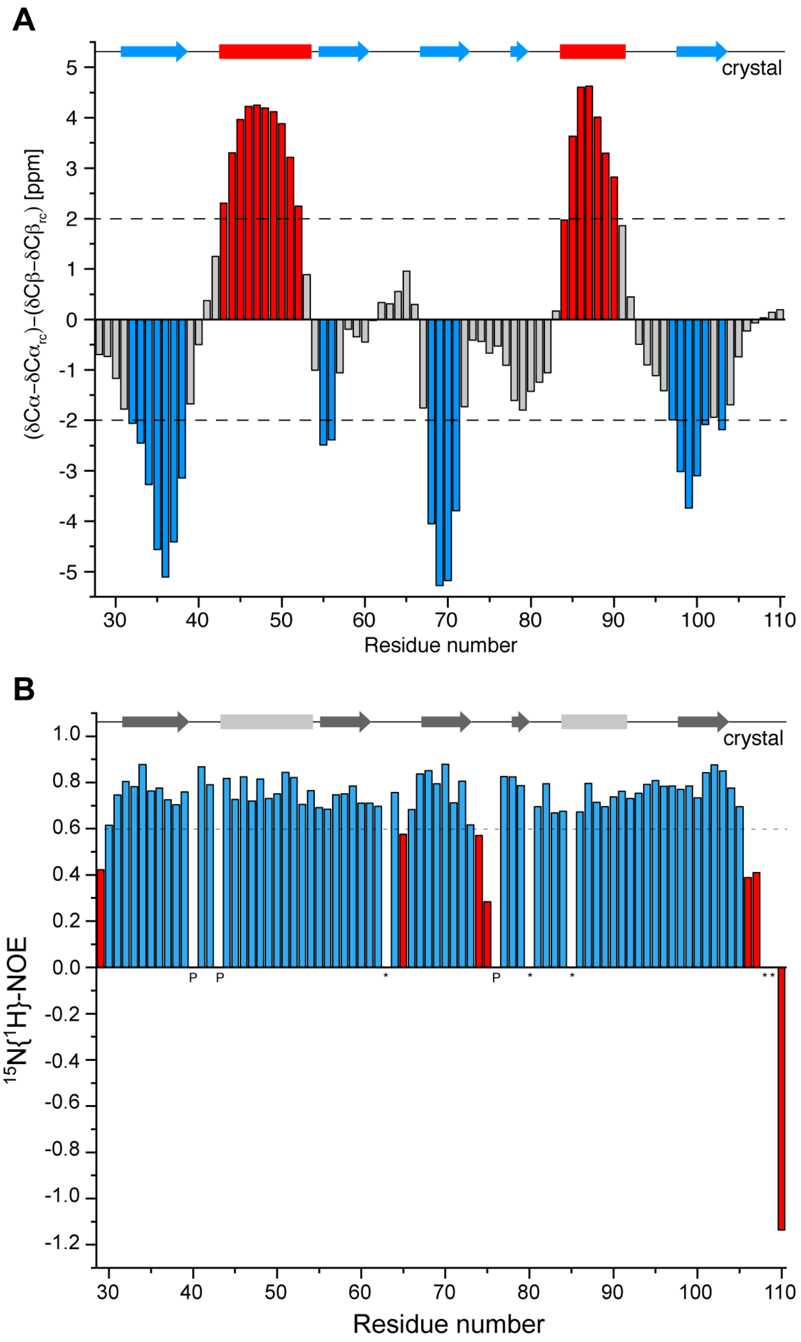

Figure S9 Secondary structure and flexibility analyses of the PC1/3-prodomain. A) Secondary structure predicted from the ^{13}C chemical shifts (n=1) of Cα and Cβ in comparison to secondary structure found in the crystal structure. Light blue bars indicate β-sheets, red bars α-helices. Data were smoothed according to (0.25*Δδ(i-1)+ 0.5*Δδ(i)+ 0.25*Δδ(i+1))/4 with i as residue number. "$_{rc}$" represents the diamagnetic random coil chemical shift. B) Steady state $^{15}N\{^{1}H\}$ nuclear Overhauser effect data (n=1) of ^{15}N-labeled PC1 prodomain measured at 298 K. Prolines that lack a detectable signal are marked with a P. Residues with missing or unassigned signals are indicated by an asterisk. Bars with values larger than 0.6 indicative of rigidity are shown in blue. Bars of flexible residues are indicated in red.

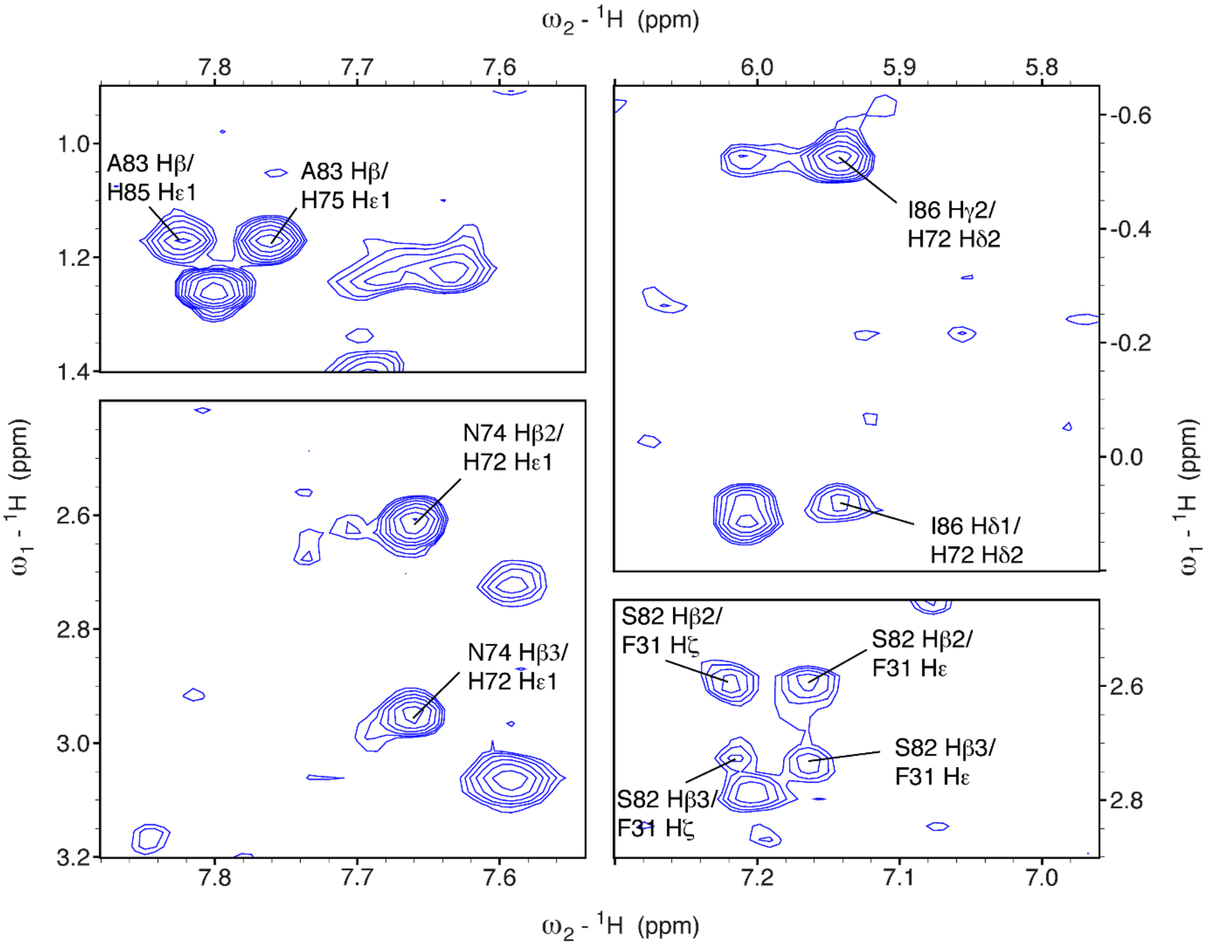

Figure S10 NMR-spectra used for NOE determinations. Regions of a 2D ^{1}H-^{1}H NOESY spectrum illustrating key NOE cross-peaks. The spectrum was recorded with a ^{15}N-labeled protein using a mixing time of 100 ms, 96 transients, 1024 × 700 complex points and ^{15}N decoupling.

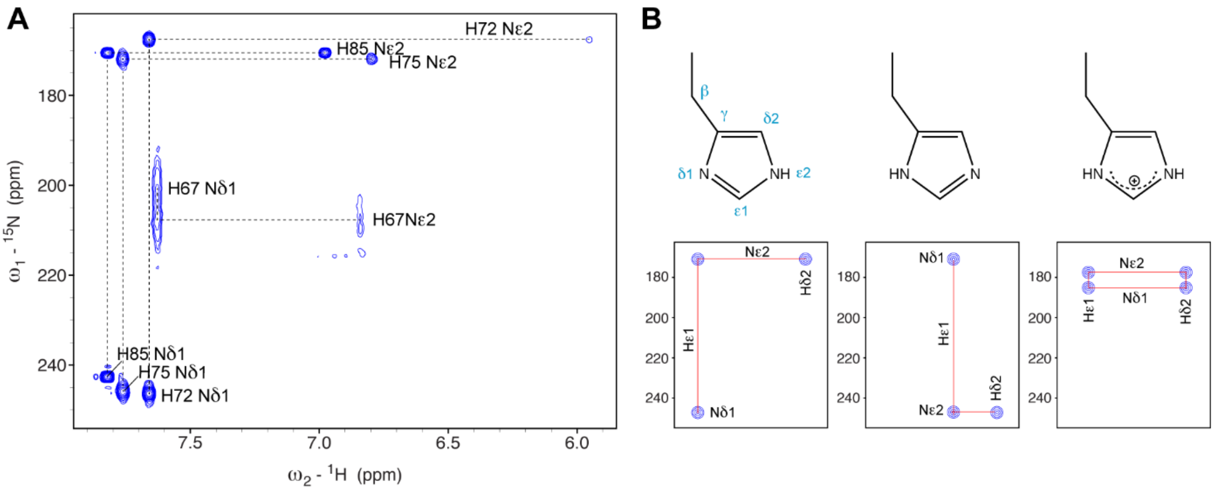

Figure S11 Tautomeric states of the histidine residues in the PC1/3-prodomain. A) 2D ^{15}N-HMBC spectrum of the PC1/3-prodomain at 298 K, pH 7.4 with assignment. B) The three possible tautomeric states of histidine side chains: the most common neutral Hε2 form (left), the less common neutral Hδ1 form (middle) and positively charged form (right) with their typical signal patterns in the ^{15}N-HMBC spectrum. His72, His75 and His85 correspond to the typical pattern of the most common Nε2H neutral tautomer. The pattern of His67 indicates a mainly positively charged side chain in fast exchange with a minor population of the neutral and rare Hδ1 form.

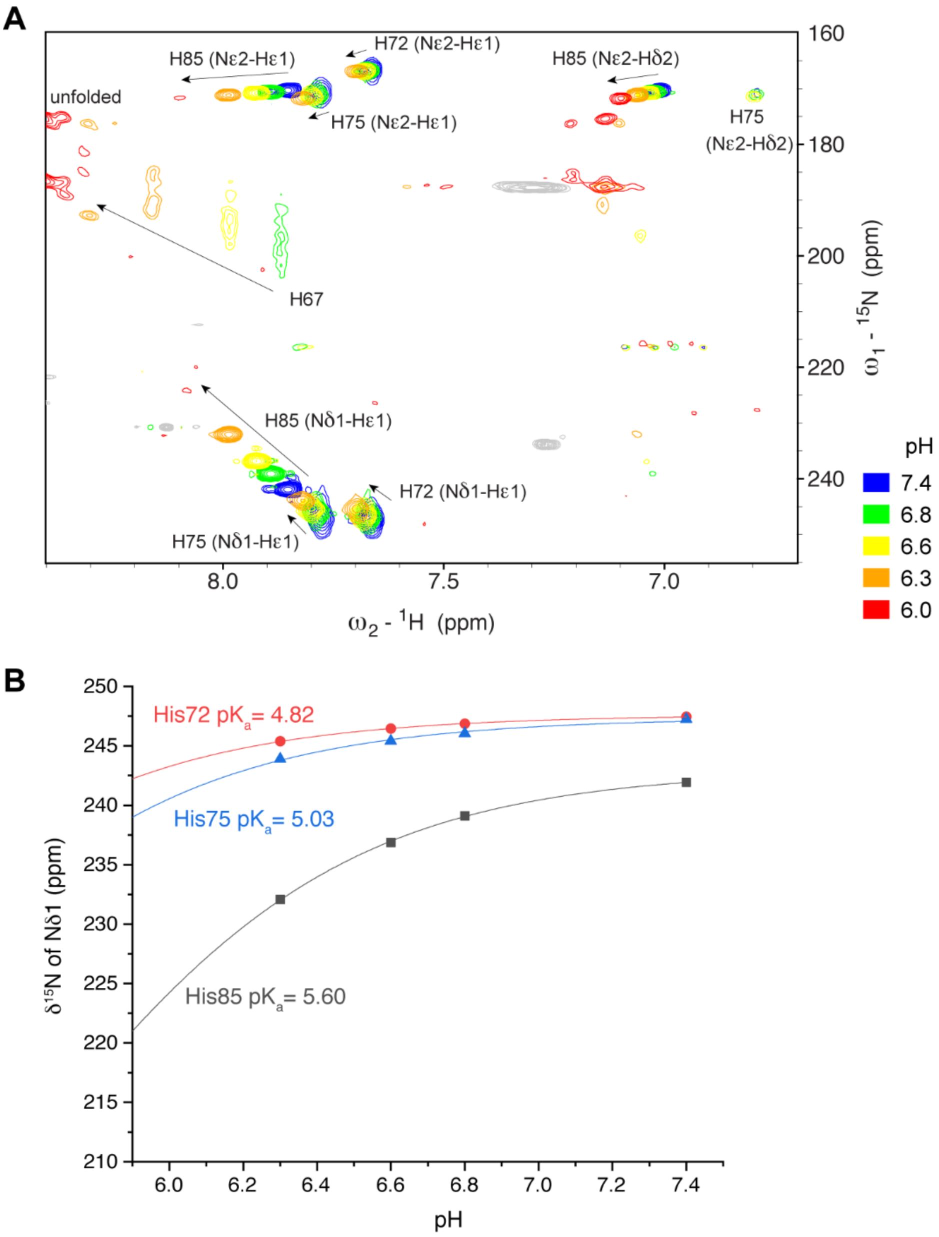

Figure S12. Estimation of pKa values of histidines in the PC1 prodomain. A) ^{1}H-^{15}N HMBC spectra of ^{15}N-PC1 prodomain at 278 K. Overlay of spectrum at pH 7.4 (blue with assignments), pH 6.8 (green), pH 6.6 (yellow), pH 6.3 (orange) and pH 6.0 (red). B) The chemical shift of His $\delta N^{\delta 1}$ as a function of pH. Data points were fitted to estimate the pKa of each histidine under the assumption that at pH 2.0 all histidine residues are protonated and that the ^{15}N chemical shift will be then 176 ppm.

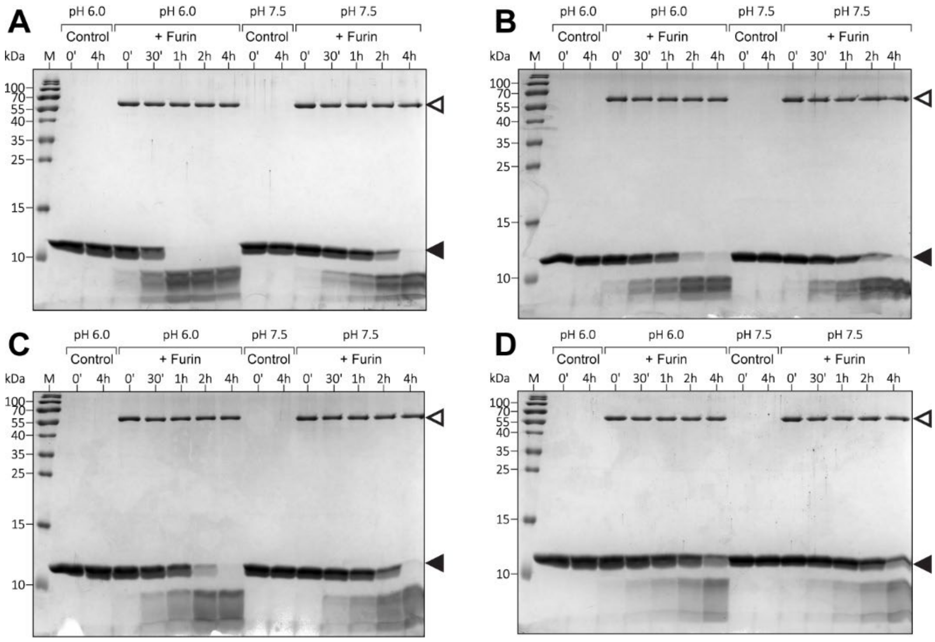

Figure S13. Limited proteolysis assays of PC1/3-prodomain H72-mutants. SDS-PAGE analysis limited proteolysis assays of A) the wild-type PC1/3-prodomain as well as of the mutants B) **M6**, C) **M7** and D) **M8**. Assays were either performed with (+Furin) or without furin (Control). The bands of furin and of the PC1/3-prodomain are highlighted by open and closed arrowheads, respectively.

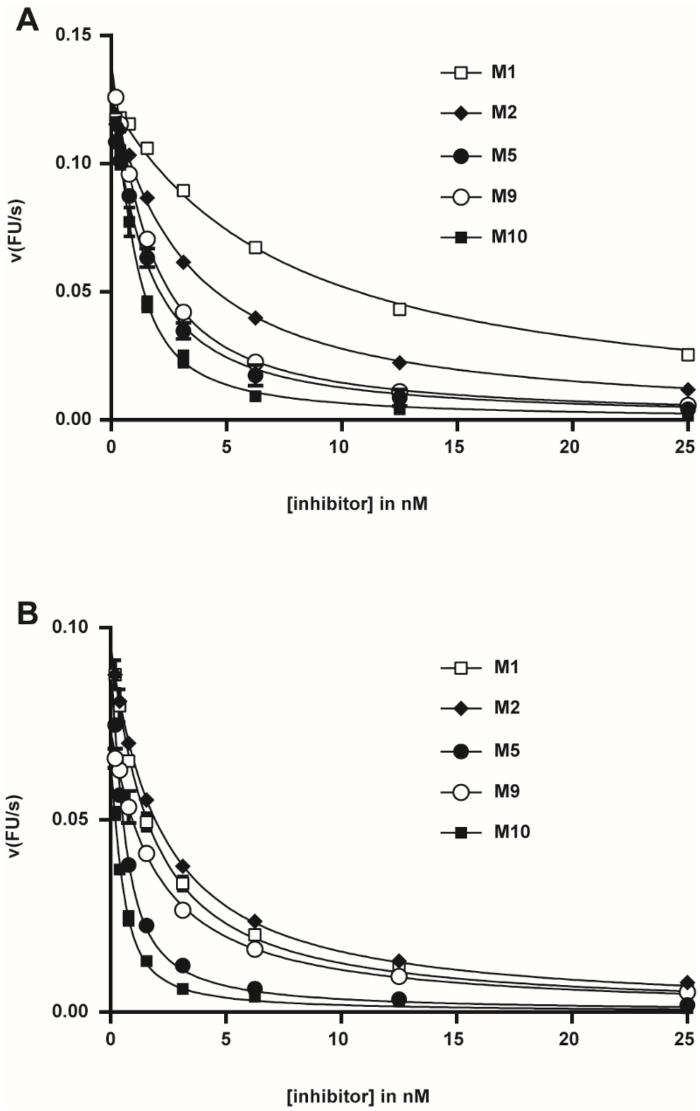

Figure S14 Inhibition of furin by PC1/3-prodomain mutants under tight binding conditions at A) pH 6.0 B) pH 7.4. The fit of the points with the Morrison equation is always shown as line. Error bars represent the standard deviations of three replicates (n=3). (M = mutant)

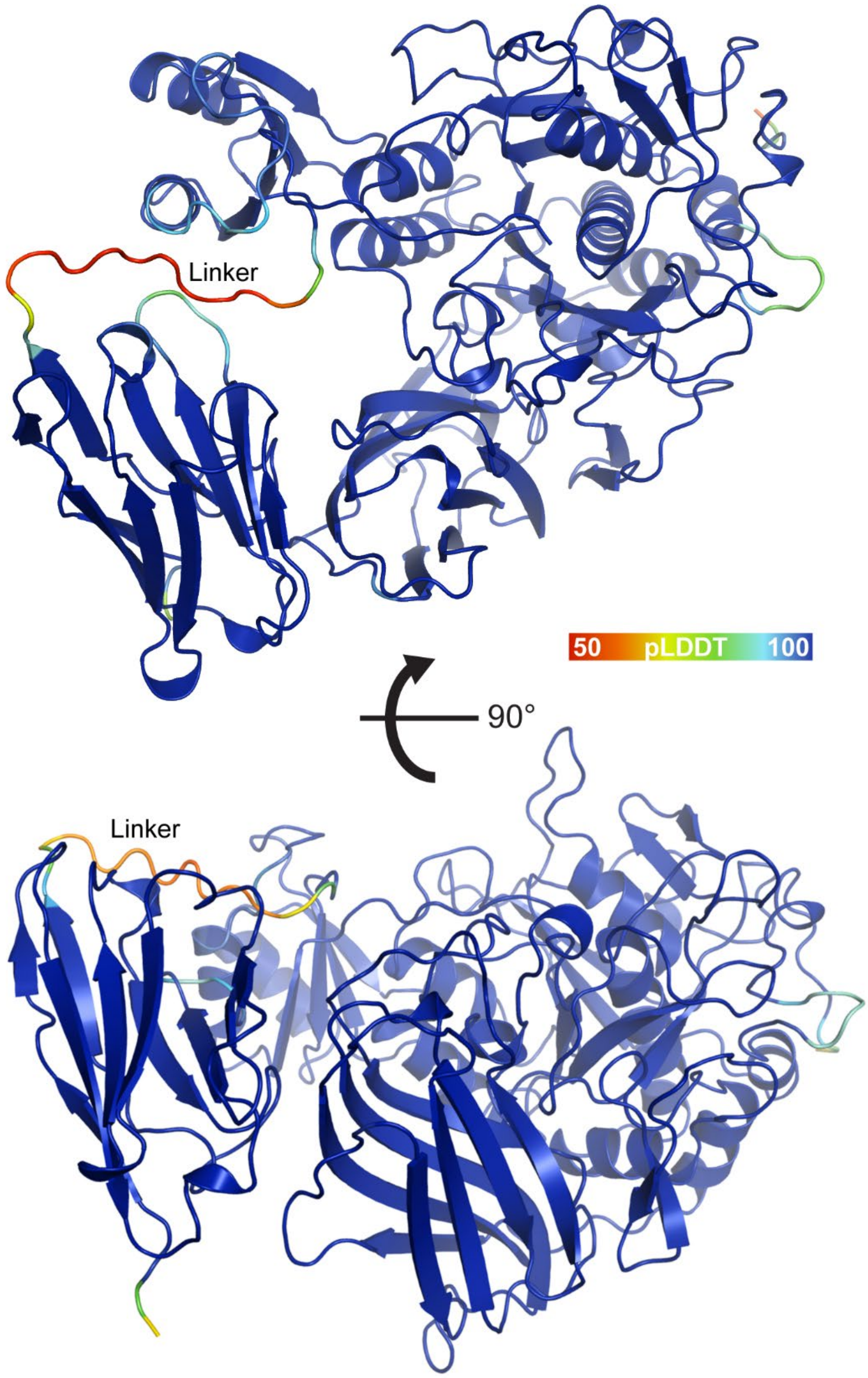

Figure S15. Predicted structure of the fusion protein **F1** in complex with furin. The confidence score (pLDDT) of the structure prediction by Alphafold 3 is shown as color gradient. Blue colors represent pLDDT values close to 100 that indicates modelling with high confidence. Red colors represent pLDDT values <50 that were modelled with low confidence. The ipTM value of the complex is 0.9. The PAE values of furin and of the fusion protein are 1.05 and 1.16, respectively.

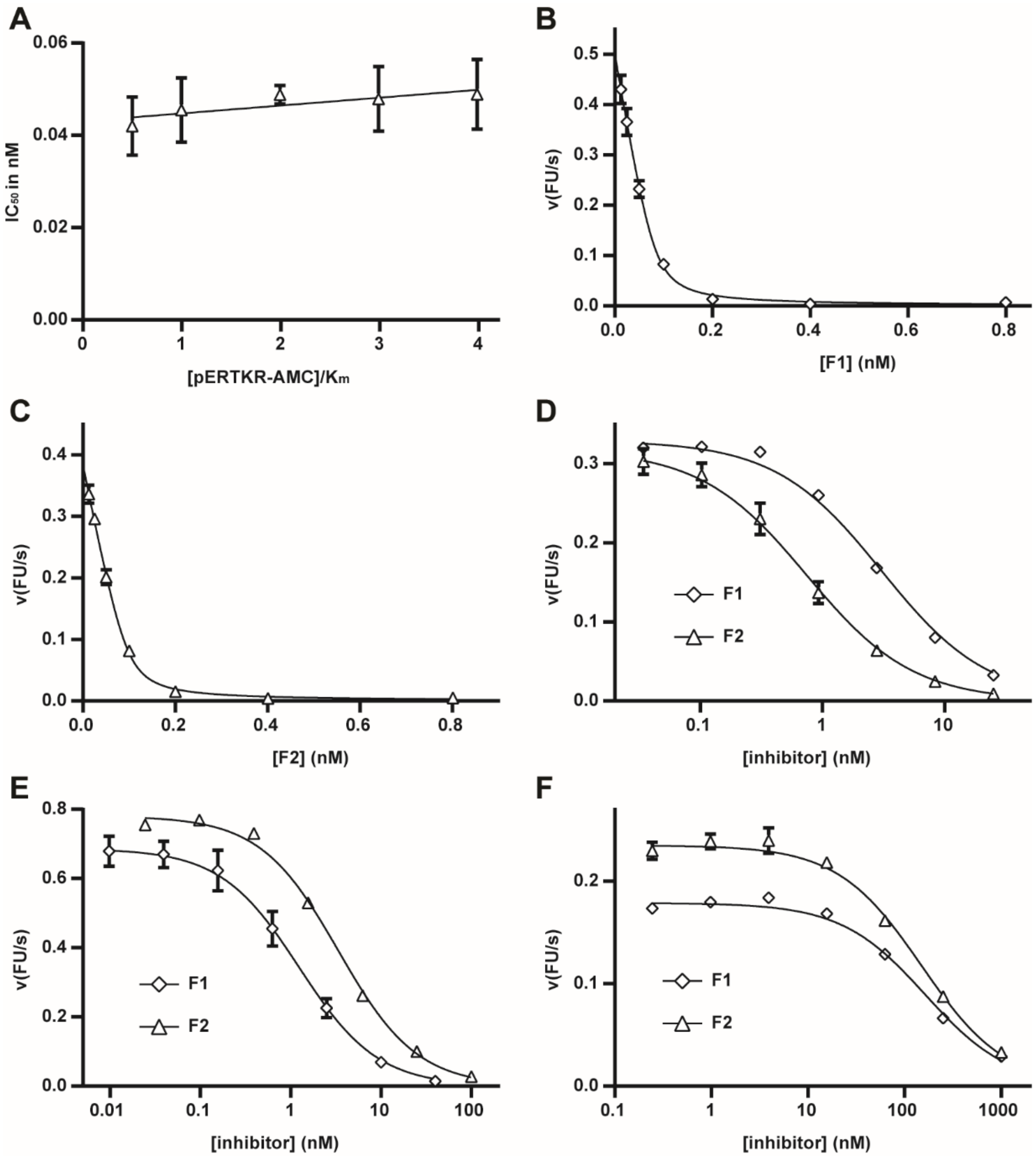

Figure S16 Specific inhibition of furin by nanobody-PC1/3-prodomain fusion proteins. A) Linear relationship of IC_{50}-values in dependence of substrate concentration/K_m indicates a competitive inhibition of furin by **F2** under tight binding conditions. The K_i value is given by the slope of the line (1.71 ± 0.66 pM). Inhibition of furin by B) **F1** and C) **F2** under tight binding conditions. The fit of the points with the Morrison equation is always shown as line. Inhibition of D) furinT562R, E) PC7 and F) PC5/6 by **F1** and **F2** fitted with an equation for competitive inhibition (line). Error bars represent the standard deviations of three replicates (n=3).

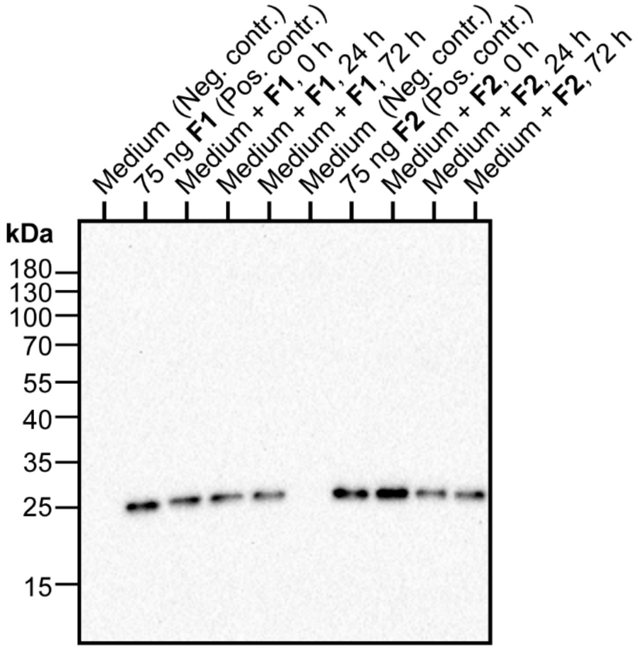

Figure S17 Detection of the fusion proteins F1 and F2 in cell culture supernatant of A549 cells after cultivation for 0-72 h. Anti-nanobody western blot using medium without the fusion proteins and the recombinant fusion proteins as positive and negative controls, respectively. The Western blot shown is representative of three experiments.

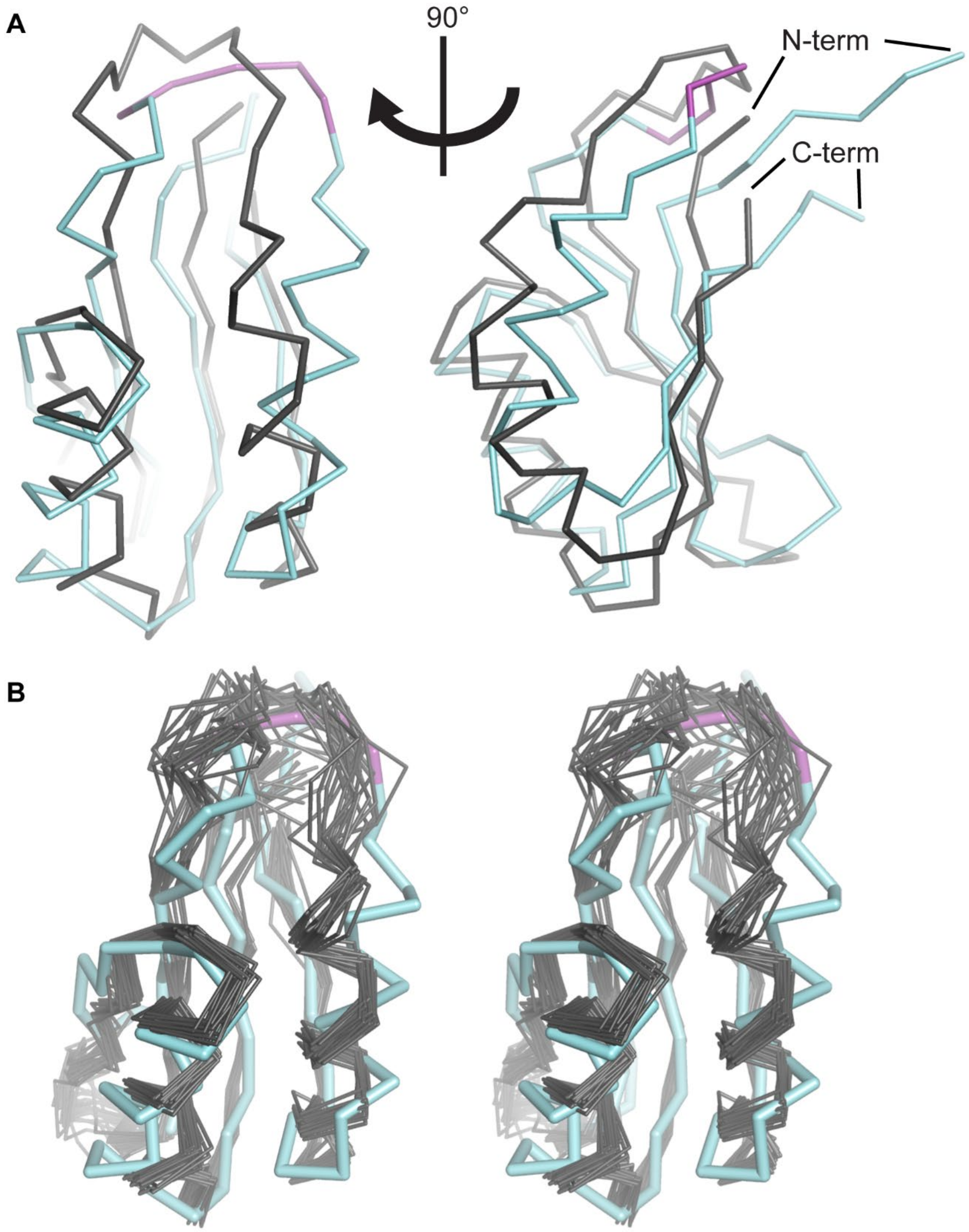

Figure S18 Alignment of the herein determined structure of isolated the PC1/3-prodomain mutant **M2** (cyan) and of the NMR-structure of the mouse PC1/3-prodomain (gray, PDB-ID 1KN6, [7]). The C_{α}-atoms of the structures are shown as ribbon representation. The secondary cleavage site loop of **M2** is highlighted in magenta. A) Two orientations of **M2** rotated by 90° as indicated. One structure (first structure in PDB-file) of the ensemble of the PDB-ID 1KN6 is shown. B) Stereo representation showing all 20 structures of the PDB-ID 1KN6.

Supplementary Tables

Table S1. Data collection and refinement statistics.

**Data collection statistics**			
PC1/3-prodomain mutant	**M5**	**M2**	**M2**
PDB ID	9FID	9FIE	9FIC
pH-value of crystallization	5.9	7.0	8.3
Number of crystals	79	5	1
Beamline	ESRF ID23-2	ESRF ID23-1	ESRF ID23-1
Wavelength	0.8731	0.8856	0.8856
Space group	$P2_1$	P1	$P6_1$
Unit cell parameters: a(Å), b (Å), c (Å)	58.5, 197.0, 99.9	82.8, 83.7, 103.2	60.0, 60.0, 40.5
α(°), β (°), γ (°)	90.0, 106.8, 90.0	98.5, 89.9, 95.9	90.0, 90.0, 120.0
Resolution range [a] (Å)	49.2-2.4 (2.46-2.40)	46.6-2.0 (2.05-2.00)	30.0-1.3 (1.38-1.30)
R_{meas} [a] (%)	53.1 (247.7)	21.0 (126.1)	9.5 (99.1)
I/sigI [a]	4.9 (1.3)	5.5 (1.2)	13.9 (2.0)
$CC_{1/2}$ (%) [a]	93.3 (23.9)	98.9 (57.8)	99.9 (77.8)
Completeness (%) [a]	75.1 (78.3)	99.7 (99.6)	100.0 (100.0)
No. of observations (total/unique)	1,291,211 / 63,282	716,591 / 183,598	207208 / 20558
Redundancy	20.4	3.9	10.1
**Refinement statistics**			
Protein molecules / asymmetric unit	6	8	1
No. of non-hydrogen atoms	12452	18219	774
Protein / water / other	12337 / 106 / 9	17315 / 871 / 33	675 / 96 / 3
R_{work}/R_{free}	22.8 / 25.2	19.7 / 21.9	14.2 / 17.2
B-factors (Å^2)			
Overall/Wilson plot	35.8/37.8	31.1/31.3	18.1 / 19.4
Protein / water / other	35.8 / 30.5 / 39.9	31.2 / 29.6 / 37.0	16.3 / 30.1 / 18.5
RMSD bond length (Å)	0.003	0.003	0.007
RMSD bonded B-factors (Å^2)	3.3	1.9	2.3

[a] Values of the highest resolution shell are given in parentheses

Table S2. Observed nuclear Overhauser effects (NOEs) in solution supporting the conformation in the crystal structure.

Observed NOESY cross-peaks		Observed cross-peak intensity	Distance in crystal structure
xx loop	close protons		[Å]
His72 Hδ2	Ile86 Hγ2	+++ [a]	2.9
His72 Hδ2	Ile86 Hδ1	+ [a]	3.3
His72 Hε1	Asn74 H^N	+ [a]	3.1
His72 Hε1	Asn74 Hβ2	+++ [a]	2.6
His72 Hε1	Asn74 Hβ3	++ [a]	4.0
His72 Hε1	His75 H^N	+ [a]	3.3
His75 Hδ2	Ile86 Hδ1	++ [a]	3.3
His75 Hε1	Ala83 Hβ	+++ [a]	2.6
Arg77 Hα	Asn33 Hβ3	+ [a]	3.1
Ser79 H^N	Phe31 H^N	+ [b]	3.2
Ser79 Hβ2	Arg81 H^N	++ [b]	3.1
Ser79 Hβ3	Arg81 H^N	+ [b]	3.9
Arg80 Hα	Phe31 H^N	+ [b]	3.3
Ser82 Hβ2	Phe31 Hε	+ [a]	2.5
Ser82 Hβ2	Phe31 Hζ	+ [a]	3.6
Ser82 Hβ3	Phe31 Hε	+ [a]	4.1
Ser82 Hβ3	Phe31 Hζ	+ [a]	5.1

[a] 2D ^{1}H-^{1}H NOESY

[b] 3D ^{15}N-edited NOESY

Table S3. Overview of enzyme kinetic measurements.

Enzyme	[Enzyme] in nM	Inhibitor[a]	Substrate[b]	[Substrate] in µM	Condition[c]	Km ± SEM used for analysis in µM	Analysis model[d]
Furin	0.5	**M1**	1	100	1	39.0 ± 2.1	1
Furin	0.5	**M2**	1	100	1	39.0 ± 2.1	1
Furin	0.5	**M3**	1	100	1	39.0 ± 2.1	1
Furin	0.5	**M4**	1	100	1	39.0 ± 2.1	1
Furin	0.5	**M5**	1	100	1	39.0 ± 2.1	1
Furin	0.05	**F1**	1	150	1	43.2 ± 1.1	1
Furin	0.05	**F2**	1	100	1	43.2 ± 1.1	1
Furin	1.0	**M1**	2	10	2	4.94 ± 0.39	1
Furin	1.0	**M2**	2	10	2	4.94 ± 0.39	1
Furin	1.0	**M5**	2	10	2	4.94 ± 0.39	1
Furin	1.0	**M9**	2	10	2	4.81 ± 0.32	1
Furin	1.0	**M10**	2	10	2	4.55 ± 0.28	1
Furin	0.5	**M1**	2	2.5	3	1.53 ± 0.05	1
Furin	0.5	**M2**	2	2.5	3	1.53 ± 0.05	1
Furin	0.5	**M5**	2	2.5	3	1.53 ± 0.05	1
Furin	0.5	**M9**	2	2.5	3	1.45 ± 0.04	1
Furin	0.5	**M10**	2	2.5	3	1.45 ± 0.07	1
Furin^{T562R}	0.05	**F1**	1	100	1	40.8 ± 1.0	2
Furin^{T562R}	0.05	**F2**	1	100	1	40.8 ± 1.0	2
PC7	0.02	**F1**	3	50	1	27.6 ± 1.1	2
PC7	0.02	**F2**	3	50	1	27.6 ± 1.1	2
PC5/6	1	**F1**	1	20	1	5.82 ± 0.51	2
PC5/6	1	**F2**	1	20	1	5.56 ± 0.23	2

[a] M = mutant, F = fusion protein

[b] Substrate 1: pERTKR-AMC, substrate 2: Ac-RR(Tle)KR-AMC (substrate 10, [8]), substrate 3: H-RR(Tle)KR-AMC (substrate 11, [8])

[c] Condition 1: 50 mM Hepes/NaOH, pH 7.4, 150 mM NaCl, 2 mM $CaCl_2$, 0.2% (v/v) Triton X-100, 37 °C; condition 2: 50 mM Mes/NaOH, pH 6.0, 300 mM NaCl, 2 mM $CaCl_2$, 0.2% (v/v) Triton X-100, 25 °C; condition 3 50 mM Hepes/NaOH, pH 7.4, 300 mM NaCl, 2 mM $CaCl_2$, 0.2% (v/v) Triton X-100, 25 °C

[d] Analysis model 1: tight binding conditions, Morrison equation; Analysis model 2: competitive inhibition: $v=V_{max}*[S]/([S]+K_m(1+[I]/K_i))$ v = reaction velocity, V_{max} = maximum velocity, [S] = substrate concentration, K_m = Michaelis-Menten constant, K_i = inhibition constant

Table S4. Experimental details of the applied NMR experiments.

Experiment	Origin [a]	Spectral widths (ppm)	Aquisition times (ms)	Number of scans	Reference
2D ^{15}N-HSQC	ETHZ	13.88 (^{1}H)	61.44	4	[9]
		34.54 (^{15}N)	121.9		
2D ^{15}N-HMBC	ETHZ	13.95 (^{1}H)	61.2	256	[10]
		102.8 (^{15}N)	10.2		
2D ^{13}C^{ali}-HSQC	ETHZ	13.95 (^{1}H)	61.2	8	[11]
		70.5 (^{13}C)	24.1		
2D ^{13}C^{aro}-HSQC	ETHZ	13.95 (^{1}H)	61.2	8	[11]
		44.17 (^{13}C)	19.2		
2D ^{15}N-^{1}H NOE [b]	ETHZ	13.89 (^{1}H)	61.4	64	[12], [13]
		34.54 (^{15}N)	47.6		
2D NOESY with ^{15}N decoupling	ETHZ	13.89 (^{1}H)	61.4	96	[c]
		12.25 (^{1}H)	47.6		
3D HNCA	ETHZ	16.02 (^{1}H)	53.2	8	[14]
		31.98 (^{13}C)	7.4		
		28.44 (^{15}N)	17.3		
3D CBCA(CO)NH	ETHZ	13.88 (^{1}H)	61.4	16	[15]
		62.04 (^{13}C)	6.51		
		28.44 (^{15}N)	19.7		
3D HNCO	ETHZ	13.88 (^{1}H)	61.4	8	[16]
		12.04 (^{13}C)	19.3		
		28.44 (^{15}N)	19.7		
3D HN(CA)CO	hncacogpwg3d from Bruker library	13.88 (^{1}H)	61.4	32	[17]
		28.44 (^{15}N)	19.7		
		12.06 (^{13}C)	22.0		
3D HNCACB	ETHZ	13.88 (^{1}H)	122.9	24	[14]
		62.05 (^{13}C)	53.4		
		28.44 (^{15}N)	19.7		
3D hCCH-TOCSY	hcchdigp3d from Bruker library	12.90 (^{1}H)	68.3	8	[18]
		66.00 (^{13}C)	53.2		
		66.00 (^{13}C)	53.2		
3D ^{13}C-edited NOESY	ETHZ	12.35 (^{1}H)	69.1	8	[d]
		37.23 (^{13}C)	8.9		
		12.35 (^{1}H)	27.0		
3D ^{15}N-edited NOESY	ETHZ	12.44 (^{1}H)	68.6	8	[19]
		34.54 (^{15}N)	14.8		
		12.44 (^{1}H)	26.8		

[a] ETHZ stands for ETH Zurich, library of the Bio-NMR facility

[b] The relaxation delay was 2 sec

[c] Implementing a ^{15}N-decoupling in a common 2D NOESY experiment is a trivial modification

[d] This version of this otherwise common experiment was not published.

Supplementary References

1 Dahms, S. O., Haider, T., Klebe, G., Steinmetzer, T. & Brandstetter, H. OFF-State-Specific Inhibition of the Proprotein Convertase Furin. *ACS Chem Biol*, doi:10.1021/acschembio.1c00411 (2021).

2 Dahms, S. O., Arciniega, M., Steinmetzer, T., Huber, R. & Than, M. E. Structure of the unliganded form of the proprotein convertase furin suggests activation by a substrate-induced mechanism. *Proc Natl Acad Sci U S A* **113**, 11196-11201, doi:10.1073/pnas.1613630113 (2016).

3 Dahms, S. O. *et al.* X-ray Structures of Human Furin in Complex with Competitive Inhibitors. *ACS Chem Biol* **9**, 1113-1118, doi:10.1021/cb500087x (2014).

4 Dahms, S. O. *et al.* The structure of a furin-antibody complex explains non-competitive inhibition by steric exclusion of substrate conformers. *Sci Rep* **6**, 34303, doi:10.1038/srep34303 (2016).

5 Aricescu, A. R., Lu, W. & Jones, E. Y. A time- and cost-efficient system for high-level protein production in mammalian cells. *Acta Crystallogr D Biol Crystallogr* **62**, 1243-1250, doi:10.1107/S0907444906029799 (2006).

6 Kuester, M. *et al.* Purification of the proprotein convertase furin by affinity chromatography based on PC-specific inhibitors. *Biol Chem* **392**, 973-981, doi:10.1515/bc.2011.100 (2011).

7 Tangrea, M. A., Bryan, P. N., Sari, N. & Orban, J. Solution structure of the pro-hormone convertase 1 pro-domain from Mus musculus. *J Mol Biol* **320**, 801-812, doi:10.1016/s0022-2836(02)00543-0 (2002).

8 Lam van, T. V. *et al.* Design, synthesis, and characterization of novel fluorogenic substrates of the proprotein convertases furin, PC1/3, PC2, PC5/6, and PC7. *Anal Biochem* **655**, 114836, doi:10.1016/j.ab.2022.114836 (2022).

9 Mori, S., Abeygunawardana, C., Johnson, M. O. & Vanzijl, P. C. M. Improved Sensitivity of HSQC Spectra of Exchanging Protons at Short Interscan Delays Using a New Fast HSQC (FHSQC) Detection Scheme That Avoids Water Saturation. *J. Magn. Reson.* **108**, 94-98, doi:10.1006/jmrb.1995.1109 (1995).

10 Bax, A., Ikura, M., Kay, L. E., Torchia, D. A. & Tschudin, R. Comparison of different modes of two-dimensional reverse-correlation NMR for the study of proteins. *J. Magn. Reson.* **86**, 304-318, doi:10.1016/0022-2364(90)90262-8 (1990).

11 Muhandiram, D. R., Farrow, N. A., Xu, G. Y., Smallcombe, S. H. & Kay, L. E. A Gradient ^{13}C NOESY-HSQC Experiment for Recording NOESY Spectra of ^{13}C-Labeled Proteins Dissolved in H_2O. *J. Magn. Reson. B* **102**, 317-321, doi:10.1006/jmrb.1993.1102 (1993).

12 Zhu, G., Xia, Y., Nicholson, L. K. & Sze, K. H. Protein dynamics measurements by TROSY-based NMR experiments. *J Magn Reson* **143**, 423-426, doi:10.1006/jmre.2000.2022 (2000).

13 Renner, C., Schleicher, M., Moroder, L. & Holak, T. A. Practical aspects of the 2D 15N-[1h]-NOE experiment. *J Biomol NMR* **23**, 23-33, doi:10.1023/a:1015385910220 (2002).

14 Grzesiek, S. & Bax, A. Improved 3D triple-resonance NMR techniques applied to a 31 kDa protein. *J. Magn. Reson.* **96**, 432-440, doi:10.1016/0022-2364(92)90099-S (1992).

15 Grzesiek, S. & Bax, A. Correlating backbone amide and side chain resonances in larger proteins by multiple relayed triple resonance NMR. *J. Am. Chem. Soc.* **114**, 6291–6293, doi:10.1021/ja00042a003 (1992).

16 Grzesiek, S., Anglister, J., Ren, H. & Bax, A. Carbon-13 line narrowing by deuterium decoupling in deuterium/carbon-13/nitrogen-15 enriched proteins. Application to triple resonance 4D J connectivity of sequential amides. *J. Am. Chem. Soc.* **115**, 4369–4370, doi:10.1021/ja00063a068 (1993).

17 Clubb, R. T., Thanabal, V. & Wagner, G. A constant-time three-dimensional triple-resonance pulse scheme to correlate intraresidue $^{1}H^{N}$, ^{15}N, and $^{13}C'$ chemical shifts in ^{15}N- ^{13}C-labelled proteins. *J. Magn. Reson.* **97**, 432-440, doi:10.1016/0022-2364(92)90252-3 (1992).

18 E., K. L., Xu, G.-Y., Singer, A. U., Muhandiram, D. R. & D., F. J. A Gradient-Enhanced HCCH-TOCSY Experiment for Recording Side-Chain ^{1}H and ^{13}C Correlations in H2O Samples of Proteins. *J. Magn. Reson. B* **101**, 333–337, doi:10.1006/jmrb.1993.1053 (1993).

19 Talluri, S. & Wagner, G. An optimized 3D NOESY-HSQC. *J Magn Reson B* **112**, 200-205, doi:10.1006/jmrb.1996.0132 (1996).
