# Supplementary material for: Structural insights into proprotein convertase activation facilitate the engineering of highly specific furin inhibitors
Source: Nat Commun. 2025 Sep 2;16:8206. doi: 10.1038/s41467-025-63479-y (PMC12405487; doi:10.1038/s41467-025-63479-y)
Supplement: Supplementary file 2 — Reporting Summary [file 41467_2025_63479_MOESM2_ESM.pdf]

nature portfolio

Corresponding author(s): Sven O. Dahms

Last updated by author(s): Aug 1, 2025

Reporting Summary

Nature Portfolio wishes to improve the reproducibility of the work that we publish. This form provides structure for consistency and transparency in reporting. For further information on Nature Portfolio policies, see our Editorial Policies and the Editorial Policy Checklist.

Statistics

For all statistical analyses, confirm that the following items are present in the figure legend, table legend, main text, or Methods section.

n/a	Confirmed	
☐	☒	The exact sample size (*n*) for each experimental group/condition, given as a discrete number and unit of measurement
☐	☒	A statement on whether measurements were taken from distinct samples or whether the same sample was measured repeatedly
☒	☐	The statistical test(s) used AND whether they are one- or two-sided<br>*Only common tests should be described solely by name; describe more complex techniques in the Methods section.*
☒	☐	A description of all covariates tested
☒	☐	A description of any assumptions or corrections, such as tests of normality and adjustment for multiple comparisons
☐	☒	A full description of the statistical parameters including central tendency (e.g. means) or other basic estimates (e.g. regression coefficient) AND variation (e.g. standard deviation) or associated estimates of uncertainty (e.g. confidence intervals)
☒	☐	For null hypothesis testing, the test statistic (e.g. *F*, *t*, *r*) with confidence intervals, effect sizes, degrees of freedom and *P* value noted<br>*Give P values as exact values whenever suitable.*
☒	☐	For Bayesian analysis, information on the choice of priors and Markov chain Monte Carlo settings
☒	☐	For hierarchical and complex designs, identification of the appropriate level for tests and full reporting of outcomes
☒	☐	Estimates of effect sizes (e.g. Cohen's *d*, Pearson's *r*), indicating how they were calculated

Our web collection on statistics for biologists contains articles on many of the points above.

Software and code

Policy information about availability of computer code

Data collection	UNICORN 5.31, MxCube 3, Tecan SparkControl V3.2, Tecan i-control 2.0.10.0, Bruker Topspin 3.5/3.6, Alphafold 3, Image Lab software 6.0.1
Data analysis	Microsoft Excel für Office 365 v 2505, Graphpad Prism 10.2.2, Adobe Photoshop CS6, XDS (v Feb 5, 2021; v Jan 10, 2022; v Jun 30, 2023), XDS-APP 2.9, CCP4 program suite 7.0.078, XSCALE (Feb 5, 2021; Jan 10, 2022), PHASER 2.8.3, COOT 0.8.8, PHENIX 1.20.1, PYMOL 2.0.7, Sparky 3.114, python3-based XSCALE processing scipt v1, tcl-based XDS processing scipt v1

For manuscripts utilizing custom algorithms or software that are central to the research but not yet described in published literature, software must be made available to editors and reviewers. We strongly encourage code deposition in a community repository (e.g. GitHub). See the Nature Portfolio guidelines for submitting code & software for further information.

Data

Policy information about availability of data

All manuscripts must include a data availability statement. This statement should provide the following information, where applicable:

- Accession codes, unique identifiers, or web links for publicly available datasets
- A description of any restrictions on data availability
- For clinical datasets or third party data, please ensure that the statement adheres to our policy

Structures of M5:furin, M2:furin and M2 are available at the protein database (PDB) under the PDB-IDs 9FID (https://doi.org/10.2210/pdb9FID/pdb), 9FIE (https://doi.org/10.2210/pdb9FIE/pdb) and 9FIC (https://doi.org/10.2210/pdb9FIC/pdb). The chemical shift assignments are made publicly available at the BioMagResBank 61 under the accession code 52871 (https://doi.org/10.13018/BMR52871).

The model of the F1:furin complex predicted with Alphafold 3, all input data used, and the complete output data are available at https://www.modelarchive.org under DOI: 10.5452/ma-mg3kp.
PDB-IDs cited in this study: 1KN6 (https://doi.org/10.2210/pdb1KN6/pdb), 5JXG (https://doi.org/10.2210/pdb5JXG/pdb).
Source data are provided with this paper.
The code of the scripts used in this work are available under https://doi.org/10.5281/zenodo.16356790.

Research involving human participants, their data, or biological material

Policy information about studies with human participants or human data. See also policy information about sex, gender (identity/presentation), and sexual orientation and race, ethnicity and racism.

Reporting on sex and gender	Does not apply to this study.
Reporting on race, ethnicity, or other socially relevant groupings	Does not apply to this study.
Population characteristics	Does not apply to this study.
Recruitment	Does not apply to this study.
Ethics oversight	Does not apply to this study.

Note that full information on the approval of the study protocol must also be provided in the manuscript.

Field-specific reporting

Please select the one below that is the best fit for your research. If you are not sure, read the appropriate sections before making your selection.

[x] Life sciences [ ] Behavioural & social sciences [ ] Ecological, evolutionary & environmental sciences

For a reference copy of the document with all sections, see nature.com/documents/nr-reporting-summary-flat.pdf

Life sciences study design

All studies must disclose on these points even when the disclosure is negative.

Sample size	No formal sample size calculation was performed in this study. Because the measurements in this study do not involve hypothesis testing, a power analysis to control statistical α- and β-errors is unnecessary. Therefore, triplicate determinations are sufficient to capture the variation (standard deviation) in the results.
Data exclusions	No data were excluded.
Replication	Measurements were performed at least in triplicates to demonstrate reproducibility. All attempts at replication were successful.
Randomization	All assays were performed with purified enzyme and substrate under identical in-vitro conditions. As there were no distinct experimental "groups" or treatment arms, formal randomization was not applicable. There are no confounding effects to be expected which could be mitigated by randomization. There are no parameters where we would expect an impact on the molecular properties of the molecules analyzed. Therefore, we cannot objectively randomize on such hypothetical parameters.
Blinding	All measurements were performed on purified enzymes and substrates under identical in-vitro conditions; no treatment or control groups were defined. Because the experimental units are molecules rather than biological subjects, there are no behavioral or physiological covariates that could introduce observer bias. Consequently, investigator blinding to group allocation is not applicable.

Reporting for specific materials, systems and methods

We require information from authors about some types of materials, experimental systems and methods used in many studies. Here, indicate whether each material, system or method listed is relevant to your study. If you are not sure if a list item applies to your research, read the appropriate section before selecting a response.

Materials & experimental systems

n/a	Involved in the study
☐	☒ Antibodies
☐	☒ Eukaryotic cell lines
☒	☐ Palaeontology and archaeology
☒	☐ Animals and other organisms
☒	☐ Clinical data
☒	☐ Dual use research of concern
☒	☐ Plants

Methods

n/a	Involved in the study
☒	☐ ChIP-seq
☒	☐ Flow cytometry
☒	☐ MRI-based neuroimaging

Antibodies

Antibodies used

In order of appearance in the manuscript:
Ab1: polyclonal rabbit serum against H7, diluted 1:500, Sino Biological Inc. (Catalog-No.: 40104-T62)
Ab2: monoclonal Anti-α-Tubulin antibody produced in mouse, diluted 1:1000, SIGMA-ALDRICH Co. (Catalog-No.: 40104-T62)
Ab3: peroxidase-conjugated secondary anti-rabbit antibody, diluted 1:6000, Agilent Dako (Catalog-No.: P021702-2)
Ab4: peroxidase-conjugated secondary anti-mouse antibody, diluted 1:6000, Agilent Dako (Catalog-No.: P026002-2)
Ab5: rabbit anti-camelid VHH, mAb cocktail, diluted 1:2000, GenScript (Catalog-No.: A02014)
Ab6: anti-rabbit peroxidase-conjugated secondary antibody, diluted 1:40000, Cell Signaling Technology (Catalog-No.: 7074S)

Validation

Ab1: Validated applications: WB, ELISA; Immunogen: Recombinant Influenza A H7N9 (A/Shanghai/1/2013) Hemagglutinin / HA Protein (Catalog#40104-V08B, Sino Biological); Source: Polyclonal Rabbit IgG
Ab2: Validated applications: "The antibody is specific for α-tubulin in immunoblotting assays ..."; Immunogen: Microtubules from chicken embryo brain; Source: Monoclonal antibody produced in mouse
Ab3: Validated Applications: ELISA; Species Reactivity: rabbit immunoglobulins of all classes; Source: Swine; Note: This secondary antibody was also used for western blot; see e.g.: Lange et al. 2024, https://doi.org/10.1002/cmdc.202400057
Ab4: Validated Applications: ELISA; Species Reactivity: all mouse IgG subclasses, mouse IgA and mouse IgM; Source: Rabbit; Note: This secondary antibody was also used for western blot; see e.g.: Lange et al. 2024, https://doi.org/10.1002/cmdc.202400057
Ab5: Validated applications: ELISA, Western Blot, Sandwich ELISA, Immunohistochemistry (IHC), Immunocytochemistry/ Immunofluorescence (ICC/IF); Immunogen: Llama VHH antibody; Source: Monoclonal Rabbit IgG cocktail
Ab6: Validated Applications: Western Blotting; Species Reactivity: anti-Rabbit; Source: Goat

Eukaryotic cell lines

Policy information about cell lines and Sex and Gender in Research

Cell line source(s)

Human embryonic kidney cells (HEK293S GnTI-, ATCC, ATCC-Number CRL-3022); Madin-Darby canine kidney cells (MDCK II, Institute of Virology, Philipps University Marburg), A549 human lung adenocarcinoma cells (Institute of Virology, Philipps-University Marburg)

Authentication

The HEK293S cell line was autheticated by the supplier. The HEK293S cell line was not authenticated at the University of Salzburg. The MDCK and A549 cell lines were authenticated years ago at the Institute of Virology (Philipps University Marburg) and were not authenticated again.

Mycoplasma contamination

HEK293S are not tested for mycoplasma contamination. All cell lines at the Institute of Virology, Marburg, are regularly tested for mycoplasma via PCR and were mycoplasma-free.

Commonly misidentified lines
(See ICLAC register)

The used cell lines are not found in the ICLAC Register of Misidentified Cell Lines.

Plants

Seed stocks

Does not apply to this study.

Novel plant genotypes

Does not apply to this study.

Authentication

Does not apply to this study.
